# Evaluation of a revised resuscitation protocol for out-of-hospital cardiac arrest patients due to COVID-19 safety protocols: a single-center retrospective study in Japan

**DOI:** 10.1038/s41598-021-92415-5

**Published:** 2021-06-21

**Authors:** Kenji Kandori, Yohei Okada, Wataru Ishii, Hiromichi Narumiya, Ryoji Iizuka

**Affiliations:** 1 Department of Emergency and Critical Care Medicine, Japanese Red Cross Society, Kyoto Daini Hospital, 355-5 Haruobicho Kamigyoku, Kyoto, 602-8026 Japan; 2 Preventive Services, School of Public Health, Kyoto University, Kyoto, Japan; 3 Department of Primary Care and Emergency Medicine, Graduate School of Medicine, Kyoto University, Kyoto, Japan

**Keywords:** Cardiovascular diseases, Risk factors

## Abstract

This study aimed to determine the association between cardiopulmonary resuscitation (CPR) under the coronavirus 2019 (COVID-19) safety protocols in our hospital and the prognosis of out-of-hospital cardiac arrest (OHCA) patients, in an urban area, where the prevalence of COVID-19 infection is relatively low. This was a single-center, retrospective, observational, cohort study conducted at a tertiary critical care center in Kyoto City, Japan. Adult OHCA patients arriving at our hospital under CPR between January 1, 2019, and December 31, 2020 were included. Our hospital implemented a revised resuscitation protocol for OHCA patients on April 1, 2020 to prevent COVID-19 transmission. This study defined the conventional CPR period as January 1, 2019 to March 31, 2020, and the COVID-19 safety protocol period as April 1, 2020 to December 31, 2020. Throughout the prehospital and in-hospital settings, resuscitation protocols about wearing personal protective equipment and airway management were revised in order to minimize the risk of infection; otherwise, the other resuscitation management had not been changed. The primary outcome was hospitalization survival. The secondary outcomes were return of spontaneous circulation after hospital arrival and 1-month survival after OHCA occurrence. The adjusted odds ratios with 95% confidence intervals (CI) were calculated for outcomes to compare the two study periods, and the multivariable logistic model was used to adjust for potential confounders. The study analyzed 443 patients, with a median age of 76 years (65–85), and included 261 men (58.9%). The percentage of hospitalization survivors during the entire research period was 16.9% (75/443 patients), with 18.7% (50/267) during the conventional CPR period and 14.2% (25/176) during the COVID-19 safety protocol period. The adjusted odds ratio for hospitalization survival during the COVID-19 safety protocol period was 0.61 (95% CI 0.32–1.18), as compared with conventional CPR. There were no cases of COVID-19 infection among the staff involved in the resuscitation in our hospital. There was no apparent difference in hospitalization survival between the OHCA patients resuscitated under the conventional CPR protocol compared with the current revised protocol for controlling COVID-19 transmission.

## Introduction

The incidence of coronavirus disease (COVID-19) continues to increase worldwide. To prevent further spreading, the American Heart Association and the European Resuscitation Council have published revised guidelines for resuscitation of out-of-hospital cardiac arrest (OHCA) patients^1,2^. During resuscitation, aerosols are potentially generated and health care providers should take precautions against COVID-19 transmission in prehospital and hospital settings. In the guidelines, wearing personal protective equipment (PPE) with an N-95 mask is especially recommended. However, there are potential disadvantages, including communication difficulties between team members and a decreased quality of the resuscitation such as chest compression procedures^3–8^. Even though the safety of health care provider is the first priority, the prognosis of OHCA patients is also an important issue. On April 1, 2020, a revised resuscitation protocol was implemented in our hospital to prevent spreading of COVID-19; however, it is unclear whether the revised protocol has affected the patients’ prognoses. If the quality of resuscitation has been compromised by the COVID-19 safety protocols and patient outcomes made worse, the protocols should be improved as much as possible. However, there are few studies that investigate the association between the precautions for COVID-19 and the outcomes of OHCA patients in areas where the prevalence of COVID-19 is relatively low^9–11^.

The aim of this study was to evaluate the association between in-hospital resuscitation under COVID-19 safety protocols, in an urban area tertiary critical care center where the prevalence of COVID-19 infection is relatively low, and the prognoses of OHCA patients.

## Methods

### Study design

This is a single-center, retrospective, observational, cohort study. The study was approved by the Clinical Research Ethics Committee of the Japanese Red Cross Society Kyoto Daini Hospital (Approval ID Sp2020-11). The Ethics Committee waived the requirement for informed consent because of the anonymous nature of the data. All procedures in this study were performed in accordance with relevant guidelines and regulations.

### Setting

This study was performed at a tertiary critical care center in Kyoto City, Japan, which is an urban area with a population of approximately 1.5 million and about 90,000 ambulance calls annually^12^. Our 672-bed hospital is one of four tertiary critical care medical centers in Kyoto City. Generally, tertiary critical care medical centers in Japan can accept emergency and critically ill patients transported by ambulance, including sepsis, acute coronary syndrome, cardiac arrest, severe trauma, and stroke patients, and can provide specialized treatment in an intensive care unit^13^. In 2019, the emergency department cases had 7610 patients who arrived by ambulance and 20,769 patients of “walk-in” status that arrived by other means^13^.

### Study population

This study included adult patients (age ≥ 18 years) with OHCA who arrived at our emergency department under CPR between January 1, 2019, and December 31, 2020. This study excluded OHCA patients who had a return of spontaneous circulation (ROSC) at hospital arrival.

### COVID-19 cases in Kyoto City

In Kyoto City, the first case of COVID-19 infection was confirmed on January 30, 2020; by the end of March, 42 cases had been confirmed. After that, the number of infections increased through mid-May; by the end of May there were 248 cases. The number of infections decreased briefly; however, since late June, the number of cases continued to increase bimodally and reached 3,369 by December 31, 2020^14^ (Supplementary Fig. 1).

### Emergency medical service resuscitation protocol in Kyoto City

Emergency medical service (EMS) basically treat the OHCA patients according to the Japanese resuscitation guidelines published from the Japan Resuscitation Council (JRC)^15^, which are developed based on the statements from the International Liaison Committee on Resuscitation (ILCOR)^16^. During the COVID-19 infection-spreading period, the EMS in Kyoto city implemented a protocol to treat all the cardiac arrest patients as possibly having COVID-19. Bag-valve mask (BVM) ventilation and chest compression were performed with attention to the fact that virus-containing aerosols might be generated. In the EMS protocol, before entering the scene, all staff donned PPE that included N95 masks and eye protection, and a high-efficiency particulate air (HEPA) filter was attached securely to any manual or mechanical ventilation device in the path of exhaled gas. Breathing was assessed by observing chest wall movement in order to minimize the risk of infection. Chest compression was started after covering the mouth and nose of the patient with a BVM and holding it close to the patient's face. Chest compression was limited to as short a time as possible when the mouth of the patient was not covered with a mask or when advanced airway management such as laryngeal tube or tracheal intubation was not introduced. It was recommended that advanced airway management be introduced as early as possible. When administering positive pressure ventilation with a BVM, EMS staff held the BVM tightly against the patient's face to minimize air leakage. Since EMS personnel are generally not allowed to terminate resuscitation in a prehospital setting, all OHCA cases were transported to a hospital.

### Revised resuscitation protocol during the COVID-19 period in our hospital

In the interest of controlling COVID-19 transmission, our hospital implemented a revised resuscitation protocol for OHCA patients on April 1, 2020. The details are described in the additional file. In brief, a restricted zone separated from other emergency beds by doors or plastic curtains was set up during resuscitation, and OHCA patients were admitted and treated only in this space (Supplementary Fig. 2). All staff involved in the resuscitation procedures were required to wear PPE, including N95 masks (Supplementary Fig. 3). An attending emergency physician was placed outside the isolated resuscitation area to direct the other team members who performed the resuscitation activities. The conventional cardiopulmonary resuscitation (CPR) period was defined as January 1, 2019 to March 31, 2020, and the COVID-19 safety protocol period was April 1, 2020 to December 31, 2020. Resuscitation was performed in accordance with JRC guidelines^15^ based on the ILCOR statements^16^. There were no major changes in resuscitation management, except for some changes related to infection control as mentioned above. No specific change in inpatient-management protocols were adopted.

### Data collection

Prehospital resuscitation data and in-hospital data were obtained by electronic chart reviews by certified emergency physician. Prehospital resuscitation data included the presence of a witness, presence of a bystander who performed CPR, initial cardiac rhythm at the scene, prehospital epinephrine administration, prehospital advanced airway management, prehospital automated external defibrillator use, the call–hospital interval, and achievement of prehospital ROSC. The call–hospital interval was defined as the period from the incoming call to the time when the patient arrived at the hospital. In-hospital data included baseline characteristics of the patients (age and sex), treatments such as coronary angiography, and use of a mechanical circulatory device (extracorporeal membrane oxygen and/or intra-aortic balloon pumping). The cause of arrest was defined as having a cardiac (e.g., acute coronary syndrome, other heart disease, presumed cardiac cause), non-cardiac (e.g., cerebrovascular diseases, respiratory diseases, malignant tumors), or an external cause (including traffic injury, fall, hanging, drowning, asphyxia, drug overdose, or any other external cause)^17,18^. The medical cause was defined as the cause of arrest other than an external cause. Patients were categorized by age as 18–64 years, 65–74 years, and ≥ 75 years. Patient outcomes were also collected.

### Outcome measures

The primary outcome of the study was survival of hospitalization, which was defined as survival at the admission to intensive care or high care unit after the resuscitation and initial evaluation and treatment. The secondary outcomes were ROSC after hospital arrival and 1-month survival after OHCA occurrence.

### Selection of variables

Based on previous studies^18–22^, six potential confounding factors were selected: age, presence of a witness, presence of bystander CPR, initial cardiac rhythm at the scene, the call–hospital interval, and the first documented cardiac rhythm at hospital arrival.

### Sample size estimation

It was estimated that at least 60–70 case outcomes would be required to account for the confounders using a logistic model, based on the generally accepted rule of 10 events per variable^23^. Considering this, it was determined that including cases from January 2019 to March 2020, before implementation of the COVID-19 safety protocols, would result in an adequate sample size for analysis.

### Statistical analysis

Data statistics for patient characteristics were calculated as a median with an interquartile range (IQR) for continuous variables and as a number with percentage for categorical variables. The crude and adjusted odds ratios (AORs) of outcomes with 95% confidence intervals (CIs) were calculated using the multivariable logistic model including all potential confounders. Missing data were not replaced or estimated. Statistical analyses were performed using JMP Pro 14 software (SAS Institute, Cary, NC, USA).

### Ethics approval and consent to participate

The Ethics Committee of Japanese Red Cross Society Kyoto Daini Hospital approved this study protocol (Sp2020-11), and the requirement of written informed consent was waived.

### Consent for publication

Not applicable.

## Results

### Patient characteristics

**Figure 1:**
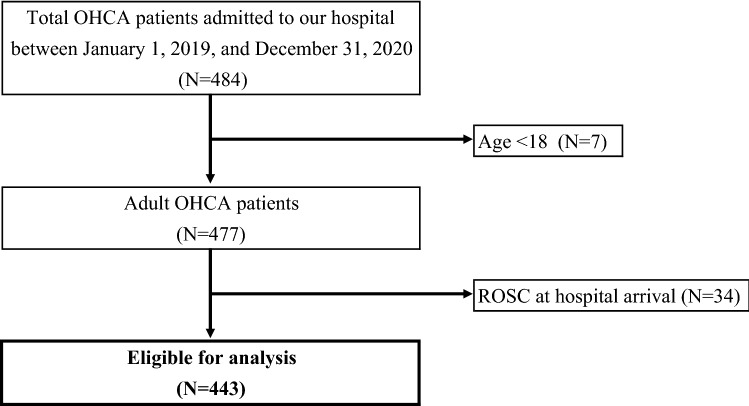
Flowchart of the study population.

Of the 484 OHCA patients admitted to our emergency department between January 1, 2019, and December 31, 2020, 7 patients aged < 18 years were excluded. Another 34 patients were excluded because they obtained ROSC at hospital arrival. The remaining 443 patients were included in the analysis (Fig. 1). Patient characteristics are shown in Table 1. In summary, the median age was 76 years (IQR 65–85), and 261 (58.9%) patients were men. The baseline characteristics were similar between the conventional and COVID-19 safety protocol period. Further, during the study period, there was no confirmed case of infected medical staff involved in a resuscitation in our hospital.

**Table 1:** Patient characteristics between the conventional CPR and COVID-19 safety protocol period.

Variables, number, (% or IQR)	All patients	The conventional CPR period (2019.01–2020.03)	The COVID-19 safety protocol period (2020.04–2020.12)
	(N = 443)	(N = 267)	(N = 176)
Age, years	76 [65–85]	77 [65–85]	76 [64–84]
**Age group, n**			
18–64 years	110 (24.8%)	65 (24.3%)	45 (25.6%)
65–74 years	87 (19.6%)	51 (19.1%)	36 (20.5%)
75 years ≤	246 (55.5%)	151 (56.6%)	95 (54.0%)
Sex, men, n	261 (58.9%)	164 (61.4%)	97 (55.1%)
**Cause of cardiac arrest, n**			
Cardiac cause	236 (53.3%)	141 (52.8%)	95 (54.0%)
Cerebrovascular cause	9 (2.0%)	8 (3.0%)	1 (0.6%)
Respiratory cause	24 (5.4%)	12 (4.5%)	12 (6.8%)
Malignant tumor	14 (3.2%)	9 (3.4%)	5 (2.8%)
External cause	135 (30.5%)	80 (30.0%)	55 (31.3%)
Others or unknown	25 (5.6%)	17 (6.4%)	8 (4.5%)
**Pre-hospital information**			
Witnessed arrest, n	160 (36.1%)	95 (35.6%)	65 (36.9%)
Bystander CPR, n	160 (36.1%)	94 (35.2%)	66 (37.5%)
**Initial cardiac rhythm at the scene**			
Shockable rhythm	35 (7.9%)	17 (6.4%)	18 (10.2%)
PEA	130 (29.3%)	85 (31.8%)	45 (25.6%)
Asystole	264 (59.6%)	155 (58.1%)	109 (61.9%)
Pre-hospital epinephrine administration, n	73 (16.5%)	53 (19.9%)	20 (11.4%)
Pre-hospital advanced airway management, n	215 (48.5%)	127 (47.6%)	88 (50.0%)
Call-hospital interval, min	28 [23–34]	28 [22–33]	29 [25–34]
**In-hospital information**			
**First documented cardiac rhythm at hospital arrival**			
Shockable rhythm	9 (2.0%)	9 (3.4%)	0 (0.0%)
PEA	121 (27.3%)	81 (30.3%)	40 (22.7%)
Asystole	313 (70.7%)	177 (66.3%)	136 (77.3%)
Tracheal intubation, n	366 (82.6%)	220 (82.4%)	146 (83.0%)
**Intervention, n**			
VA-ECMO	25 (5.6%)	15 (5.6%)	10 (5.7%)
IABP	17 (3.8%)	12 (4.5%)	5 (2.8%)
CAG	23 (5.2%)	16 (6.0%)	7 (4.0%)
PCI	11 (2.5%)	8 (3.0%)	3 (1.7%)
TTM	27 (6.1%)	17 (6.4%)	10 (5.7%)

Values are median (interquartile range [IQR]) or number (percentage).

*CAG* coronary angiography, *COVID-19* coronavirus 2019, *CPR* cardiopulmonary resuscitation, *IABP* intra-aortic balloon pumping, *IQR* interquartile range, *OHCA* out-of-hospital cardiac arrest, *PCI* percutaneous coronary intervention, *PEA* pulseless electrical activity, *ROSC* return of spontaneous circulation, *TTM* targeted temperature management, *VA-ECMO* veno-arterial extracorporeal membrane oxygenation.

### Outcomes

The primary and secondary outcomes are shown in Table 2. As primary outcome, the percentage of hospitalization after ROSC in the entire research period was 16.9% (75/443 patients). During the conventional CPR period (January 2019–March 2020), it was 18.7% (50/267) compared with 14.2% (25/176) for the COVID-19 safety protocol period (April–December 2020).

**Table 2:** The survival outcomes and multivariable logistic regression analysis for outcomes during the COVID-19 safety protocol period.

Outcomes, number, (%)	All patients	The conventional CPR period	The COVID-19 safety protocol period	The COVID-19 safety protocol	
	(N = 443)	(2019.01–2020.03) (N = 267)	(2020.04–2020.12) (N = 176)	Crude OR [95% CI]	Adjusted OR [95% CI]
Hospitalization survival	75 (16.9%)	50 (18.7%)	25 (14.2%)	0.72 [0.43–1.21]	0.61 [0.32–1.18]
ROSC after hospital arrival	152 (34.3%)	89 (33.3%)	63 (35.8%)	1.12 [0.75–1.66]	1.11 [0.69–1.79]
1-month survival after OHCA	23 (5.2%)	14 (5.2%)	9 (5.1%)	0.97 [0.41–2.30]	1.14 [0.37–3.50]

Values are number (percentage). Confounding variables included resuscitation under the COVID-19 safety protocol, age, presence of witness, presence of bystander CPR (cardiopulmonary resuscitation), initial cardiac rhythm at the scene, call–hospital interval, and the first documented cardiac rhythm at hospital arrival.

*CI* confidence interval, *COVID-19* coronavirus 2019, *OHCA* out-of-hospital cardiac arrest, *OR* odds ratio, *ROSC* return of spontaneous circulation.

As a secondary outcome, the incidence of ROSC after hospital arrival during the entire research period was 34.3% (152/443 patients); during the conventional CPR period, it was 33.3% (89/267), as compared with 35.8% (63/176) for the COVID-19 infection safety protocol period. The rate of 1-month survival after OHCA occurrence during the entire research period was 5.2% (23/443 patients); during the conventional CPR period, it was 5.2% (14/267), as compared with 5.1% (9/176) for the COVID-19 safety protocol period.

### Primary analysis

For the primary analysis, multivariable logistic regression analysis showed that the AOR for hospitalization survival during the COVID-19 safety protocol period was 0.61 (95% CI 0.32–1.18), as compared with conventional CPR (Table 2). The AORs of other confounders are shown in Supplementary Table 1.

For the secondary outcomes, multivariable logistic regression analysis showed that the AOR for ROSC after hospital arrival during the COVID-19 safety protocol period was 1.11 (95% CI 0.69–1.79), as compared with conventional CPR (Table 2). For 1-month survival, the AOR of the COVID-19 safety protocol period was 1.14 (95% CI 0.37–3.50), as compared with conventional CPR (Table 2).

## Discussion

### Key observations

The study results suggest that there was no difference in the survival outcomes between OHCA patients resuscitated under the current COVID-19 safety protocol compared with those treated before the protocol was implemented. This suggests that CPR under the current infection control measures for COVID-19 is be able to ensure the quality of resuscitation, and is acceptable for continuance. Furthermore, there were no cases of infection among the staff involved in the resuscitation in our hospital, suggesting that their safety is also accounted for.

### Strengths of the study

One strength of this study is its setting in a non-pandemic area. The majority of previous studies on OHCA were conducted in COVID-19 pandemic areas^24–36^. Most of these studies reported that OHCA resuscitation attempted at the scene declined during the COVID-19 pandemic^24–26,28–34^. Therefore, there is a limitation to applying the results of studies in pandemic regions to non-pandemic regions. On the other hand, some of the regions have not reached, or are recovering from, a COVID-19 pandemic. For such a non-pandemic setting, the result in this study may be valuable.

Second, this is the first study to focus on patient outcomes before and after the in-hospital resuscitation protocol changes due to COVID-19 safety protocols among OHCA patients. Most previous studies focused on prehospital settings such as arrest witnesses and bystander CPR^9–11,24–33,35^. However, in-hospital resuscitation has been also changing due to COVID-19 safety protocols, which may limit the number of personnel involved in resuscitation, make communication more difficult, and increase the physical and psychological burden compared to conventional resuscitation^1–3,8^. A previous study indicated that percutaneous coronary intervention was withheld even after transport to the hospital^36^. Therefore, it was hypothesized that changes in in-hospital resuscitation might potentially result in poor prognoses of OHCA patients, and it is important to examine the association between the prognoses of OHCA patients and in-hospital resuscitation under COVID-19 safety protocols. However, the study result did not support the hypothesis. Because resuscitation under COVID-19 safety protocols is expected to continue in the future, this result may be helpful when considering in-hospital resuscitation strategies.

The third strength of our study is that the risk of recruitment bias is assumed as minimum, because termination of CPR by EMS is basically not allowed. Previous studies have reported that termination of CPR at the scene increased during the pandemic^9,29–31,34^. Further, the number of resuscitation attempts at the scene was reported to decrease^24,26,28,30–35^. Among these cases, the cases transferred to the hospital might be selected as likely to obtain good outcomes. Conversely, in Japan, EMS personnel are generally not allowed to terminate resuscitation in a prehospital setting, almost all OHCA cases are transported to hospitals.

The forth strength of this study is that the results may be valid due to the design being considered a kind of natural experiment. A natural experiment is defined as a way to assess the effect of interventions or policy changes for which planned and controlled experimental research designs may be infeasible or inappropriate to implement^13,37,38^. Similar to a randomized controlled trial, the approach to this study has a strength in that the patient's background and treatment can be considered as equipoise before and after the intervention, and that the effects of unmeasured confounding may be less pronounced. Therefore, this type of study design has attracted interest as an alternative to a randomized controlled trial^38^. Since a randomized trial on resuscitation under COVID-19 safety protocols is neither practical nor ethically feasible to conduct, this current study, the effect of COVID-19 safety protocols on resuscitation could be evaluated as if it was an experiment, albeit not under control. Throughout the prehospital and in-hospital management of OHCA patients, the resuscitation protocol was only changed at the point of infection control, not otherwise. Furthermore, since the area in this study period was considered to be less affected by COVID-19 infection, because the number of COVID-19 patients was limited; thus, the patients’ background after the revised protocol was considered almost same as before. Therefore, the presence or absence of the COVID-19 safety protocol can be considered the only variable changed in the two cohorts, and the effect of unmeasured confounding is likely to be small. In this regard, the results of this study should be highly valid.

### Interpretation of the results

The results of this study suggest that resuscitation under COVID-19 safety protocols do not strongly affect the prognoses of OHCA patients. There are some possible reasons for this. The first is the proficiency of resuscitation wearing PPE. In our emergency room, health care providers wear the PPE not only in resuscitation but also when treating patients with possible COVID-19 infection such as those with fever. In addition, when performing tracheal intubation, the procedure is always performed under PPE that includes N95 masks. Therefore, health care providers became accustomed to performing medical treatments and procedures under the COVID-19 safety protocols, which may help retain the quality of resuscitation.

Secondly, in all cases, attending emergency physicians are placed outside the restricted zone and resuscitation is performed under their supervision; this could minimize the confusion at the resuscitation area and sustain the quality of CPR. Generally, the actual scene of resuscitation is sometimes chaotic under normal conditions. Further, the difficulty in communicating among the members due to the wearing of PPE, including N95 masks, may lead to losing the necessary command and control. In addition, even if the medical staff is familiar with PPE, decision-making for the resuscitation strategy in a stressful situation wearing PPE might cause a high degree of physical and mental fatigue. This might threaten not only the quality of resuscitation but also the safety of the staff. In our hospital, the attending emergency physician directs the resuscitation and makes decisions outside the isolation room while keeping an eye on the safety of the staff performing the CPR. This enables the team members to focus on the procedures while attending to their own safety. This may suggest that it is possible to guarantee the quality of resuscitation while ensuring safety.

### Limitations

This study has several limitations. First, the sample size was limited and the statistical power might be inadequate to detect differences in the outcomes. Even though this limitation is understood, the study was necessary because if resuscitation under the current safety protocol had led to significantly worse poor outcomes, immediate improvements would have been needed. Further verification with a larger sample size is needed. Second, although this study is like as a natural experiment, some potential unmeasured confounders might influence the results. Third, regarding the single-center study design, the generalizability of these results to another hospital is unclear. Fourth, the results may also vary in our hospital if the study period is different. If the number of COVID-19 cases increases dramatically in the future, the prognoses may be poor, as in reported pandemic areas. Furthermore, because a polymerase chain reaction test was not performed for all OHCA patients, the prevalence of COVID-19 with OHCA patients included in this study was unknown. Therefore, it is impossible to determine whether our COVID-19 safety protocols were perfect for infection control.

## Conclusions

This study showed that there is no significant change in hospitalization survival outcomes between OHCA patients treated by conventional CPR and those treated under the current measures for controlling COVID-19 transmission.

## Supplementary Information

Supplementary Information.

## Data Availability

Not applicable.

## Abbreviations

AOR: Adjusted odds ratio
BVM: Bag-valve mask
CI: Confidence interval
COVID-19: Coronavirus disease
CPR: Cardiopulmonary resuscitation
EMS: Emergency medical service
HEPA: High-efficiency particulate air
ILCOR: International Liaison Committee on Resuscitation
IQR: Interquartile range
JRC: Japan Resuscitation Council
OHCA: Out-of-hospital cardiac arrest
PPE: Personal protective equipment
ROSC: Return of spontaneous circulation
