# Supplementary material for: Evaluation of a revised resuscitation protocol for out-of-hospital cardiac arrest patients due to COVID-19 safety protocols: a single-center retrospective study in Japan
Source: Sci Rep. 2021 Jun 21;11:12985. doi: 10.1038/s41598-021-92415-5 (PMC8217508; doi:10.1038/s41598-021-92415-5)
Supplement: Supplementary file 1 — Supplementary Information. [file 41598_2021_92415_MOESM1_ESM.docx]

**Additional file**

**Title:** Evaluation of a revised resuscitation protocol for out-of-hospital cardiac arrest patients due to COVID-19 safety protocols: A single-center retrospective study in Japan

**Authors**

Kenji Kandori^1^, Yohei Okada^2,3^, Wataru Ishii^1^, Hiromichi Narumiya^1^, Ryoji Iizuka^1^

**Contents**

| **Supplementary Appendix 1.** | Resuscitation protocol under COVID-19 safety protocols in our hospital |
| --- | --- |
| **Supplementary Figure 1.** | The number of COVID-19 patients in Kyoto City |
| **Supplementary Figure 2.** | Flow of accepting OHCA patients in our emergency department |
| **Supplementary Figure 3.** | The PPE for resuscitation in our hospital |
| **Supplementary Table 1.** | Multivariable logistic regression analysis for hospitalization survival |

**Supplementary Appendix 1. Resuscitation protocol under COVID-19 safety protocols in our hospital**

Our hospital implemented a revised resuscitation protocol for out-of-hospital cardiac arrest (OHCA) patients on April 1, 2020 to prevent spreading coronavirus 2019 (COVID-19) in-hospital infection. All OHCA patients are handled as possible COVID-19 infections based on the guidelines in which cardiopulmonary resuscitation (CPR) have the potential to generate aerosols {Perkins, 2020, International Liaison Committee on Resuscitation: COVID-19 consensus on science`, treatment recommendations and task force insights}(1-3).

**Resuscitation area**

OHCA patients were admitted only to an area separated from other emergency beds by doors or plastic curtains. A restricted zone was set up and nobody was allowed to enter the area without appropriate personal protective equipment (PPE) during resuscitation. There are two beds for this management in the emergency department in our hospital (**Supplementary figure 2**).

**Resuscitation staff and equipment**

The number of staff in the room or on the scene was limited to only those essential for patient care. All staff involved in resuscitation procedures were required to wear PPE, including N95 masks (**Supplementary figure 3**). An attending emergency physician is placed outside the resuscitation area to direct the treatment strategy, and the other team members perform resuscitation under their guidance.

**Resuscitation procedure**

If the emergency medical service (EMS) has not introduced advanced airway management such as laryngeal tube (LT) or tracheal intubation for OHCA patients in the prehospital setting, chest compressions is interrupted between the time of hospital arrival and when the patient is placed in an isolation resuscitation room in order to prevent generating aerosol due to chest compression and ventilation. Cardiac rhythm confirmation was performed immediately after the patient was transferred to a bed in the resuscitation room. Resuscitation staff immediately implement tracheal intubation under video laryngoscopy, attach a high-efficiency particulate air (HEPA) filter and a portable end-tidal carbon dioxide monitor. Then, they resume chest compression and ventilation. For OHCA patients in whom LT has already been implemented in the prehospital setting, resuscitation staff continue chest compressions and place the patient in an isolation resuscitation room. After that, chest compressions are interrupted, the LT tube is removed, tracheal intubation is performed as in the procedure described above, and chest compressions are resumed. All resuscitation procedures and policies are carried out under the direction of the physician stationed outside the isolation resuscitation room. The decision to terminate resuscitation is also made by the physician.

**Supplementary Figure 1. The number of COVID-19 patients in Kyoto City**

The line chart shows the number of COVID-19 patients in Kyoto City per day. The first case of COVID-19 was confirmed on January 30, 2020. After that, the number of infected patients increased through mid-May. The number of infected patients decreased briefly; but, starting in late June, the number of infected patients has increased bimodally.

**Supplementary Figure 2. Flow of accepting OHCA patients in our emergency department**


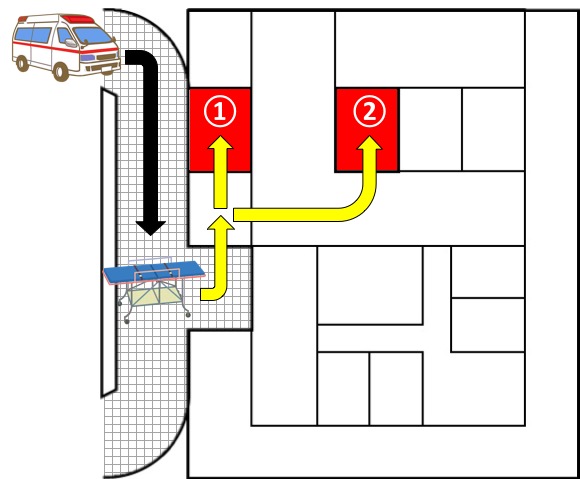


The resuscitation rooms were set up close to the ambulance loading dock. OHCA patients were admitted only to the area separated from other emergency beds by doors or plastic curtains. There were two beds (① , ② ) for this management in the emergency department in our hospital. If the EMS had not introduced advanced airway management in the prehospital setting, chest compressions were interrupted between the hospital arrival time and when the patient was placed in an isolated resuscitation room.

**Supplementary Figure 3. The PPE for resuscitation in our hospital**


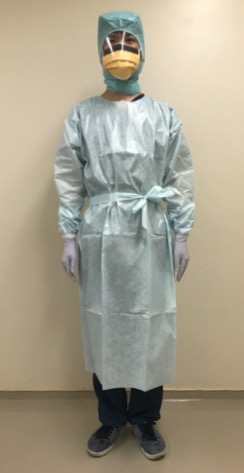


All staff involved in resuscitation procedures was required to wear PPE.

The PPE for resuscitation in our hospital comprises:

• Double gloves

• Long-sleeved isolation gowns

• Double masks: an N95 mask and a surgical mask with eye protection

• Disposable surgical caps

**Supplementary Table 1.** **Multivariable logistic regression analysis for hospitalization survival**

| **Variables** | | **n/N** | **(%)** | **Crude OR [95%CI]** | **Adjusted OR [95%CI]** |
| --- | --- | --- | --- | --- | --- |
| **Age** | |  |  |  |  |
|  | **65–74 years** | 17/87 | (19.5) | 0.65 [0.33–1.27] | 0.68 [0.30–1.57] |
|  | **75 years ≤** | 28/246 | (11.4) | 0.34 [0.19–0.61] | 0.38 [0.19–0.75] |
| **Presence of witness (Yes)** | | 54/160 | (33.8) | 6.33 [3.64–11.00] | 4.5 [2.42–8.39] |
| **Presence of bystander CPR (Yes)** | | 44/160 | (27.5) | 3.07 [1.85–5.11] | 2.3 [1.24–4.28] |
| **Initial cardiac rhythm at the scene** | |  |  |  |  |
|  | **Non-shockable rhythm** | 49/394 | (12.4) | Reference | Reference |
|  | **Shockable rhythm** | 23/35 | (65.7) | 13.49 [6.31–28.84] | 9.49 [3.67–24.55] |
| **Call–hospital interval** | |  |  |  |  |
|  | **28 minutes ≤** | 26/239 | (10.9) | 0.38 [0.23–0.64] | 0.37 [0.20–0.70] |
| **The first documented cardiac rhythm at hospital arrival** | |  |  |  |  |
|  | **Non-shockable rhythm** | 68/434 | (15.7) | Reference | Reference |
|  | **Shockable rhythm** | 7/9 | (77.8) | 18.84 [3.83–92.62] | 8.68 [1.11–68.18] |
| **The COVID-19 safety protocol (Yes)** | | 25/176 | (14.2) | 0.72 [0.43–1.21] | 0.61 [0.32–1.18] |

Confounding variables included resuscitation under the COVID-19 safety protocol, age, presence of witness, presence of bystander CPR, initial cardiac rhythm at the scene, call–hospital interval, and the first documented cardiac rhythm at hospital arrival. CI, confidence interval; COVID-19, coronavirus 2019; CPR, cardiopulmonary resuscitation; OR, odds ratio

**References**

1. Perkins GD, Morley PT, Nolan JP, Soar J, Berg K, Olasveengen T, et al. International Liaison Committee on Resuscitation: COVID-19 consensus on science, treatment recommendations and task force insights. Resuscitation. 2020;151:145–7.

2. Edelson DP, Sasson C, Chan PS, et al. Interim guidance for basic and advanced life support in adults, children, and neonates with suspected or confirmed COVID-19: From the Emergency Cardiovascular Care Committee and Get With The Guidelines-Resuscitation Adult and Pediatric Task Forces of the American Heart Association. Circulation. 2020;141:e933-e43.

3. Nolan JP, Monsieurs KG, Bossaert L, et al. European Resuscitation Council COVID-19 guidelines executive summary. Resuscitation. 2020;153:45–55.
